# Metabolomics of Clinical Poisoning by *Aconitum* Alkaloids Using Derivatization LC-MS

**DOI:** 10.3389/fphar.2019.00275

**Published:** 2019-03-22

**Authors:** Yida Zhang, Xiqing Bian, Jing Yang, Haiying Wu, Jian-Lin Wu, Na Li

**Affiliations:** ^1^State Key Laboratory of Quality Research in Chinese Medicine, Macau Institute for Applied Research in Medicine and Health, Macau University of Science and Technology, Macao, China; ^2^Department of Emergency, The First Affiliated Hospital of Kunming Medical University, Kunming, China

**Keywords:** metabolomics, clinical poisoning, *Aconitum kusnezoffii*, derivatization, carboxyl-containing metabolites, metabolic pathway

## Abstract

The root of *Aconitum kusnezoffii* (Caowu in Chinese, CW) is not only commonly used as a traditional Chinese medicine (TCM), but also served as a tonic in China. Due to its high toxicity, clinical poisoning cases induced by CW have frequently been reported. However, the mechanism is still unclear. In this study, *Aconitum* alkaloids and altered endogenous metabolites in CW poisoning patients were investigated to elucidate the possible intoxication mechanism. Eighteen alkaloids, including 6 toxic diester diterpenoid alkaloids (DDAs), were determined from the sera of patients. At the same time, 5-(diisopropylamino)amylamine (DIAAA) derivatization-ultrahigh performance liquid chromatography- quadrupole-time of flight mass spectrometry (UHPLC-Q-TOF/MS) approach was applied in the metabolomics analysis to find much more carboxyl-containing metabolites (CCMs), which are the essential components for life and critical to elucidate the mechanism of toxicity. As a result, 32 altered metabolites after poisoning were identified. Among them, hydroxyeicosatetraenoic acids (HETEs) and some dicarboxylic acids were first found to be related to *Aconitum* alkaloids toxicity. Finally, biological pathway analysis indicated that the significantly changed metabolites were primarily involved in amino acid metabolism, TCA cycle, fatty acid metabolism, pyruvate metabolism, arachidonic acid metabolism, sphingolipid metabolism and so on. These results can not only provide more information on the mechanism of CW intoxication but also help the clinical diagnosis of CW poisoning.

## Introduction

*Aconitum* species have been used as important sources for traditional Chinese medicine (TCM) in China for over 2000 years. The processed root of *Aconitum kusnezoffii* (Caowu in Chinese, CW) is commonly used to treat various diseases or deal with poor health conditions, such as syncope, rheumatic fever, painful joints, gastroenteritis, diarrhea, oedema, bronchial asthma, and some endocrine disorders (Liu et al., 2011). The major active components in CW are a series of diterpene alkaloids, such as aconitine (A), mesaconitine (MA), and hypaconitine (HA), which also have strong neurotoxicity and cardiotoxicity. People would be poisoned by consumption of only 3 to 4.5 g CW extracts (Lin et al., 2004; Singhuber et al., 2009; Chan, 2011). More seriously, its toxicity is not paid enough attention in China, especially the southwestern area, where local people usually consume it as a vegetable or prepare herbal soups and meals for improving health (Kang et al., 2012). In addition, in northern China, people who suffer from arthritis caused by cold weather often take CW to treat disease or relieve the pain (Li et al., 2016). Due to its high toxicity and widespread usage, the poisoning cases induced by CW have frequently been reported in China (Singhuber et al., 2009; Liu et al., 2011; Li et al., 2016). However, just like the huge challenges in front of all toxic TCMs, the mechanism of the toxicity of CW is still ambiguous. Until now, there is still a lack of clinical information on metabolic regulation or specific biomarkers for the diagnosis of *Aconitum* toxic alkaloids poisoning.

Metabolomics is considered to be the best strategy that can be used to understand the global metabolic regulation of how a living system response to xenobiotic stimuli, e.g., drugs and toxins (Wang et al., 2012). For now, the potential toxic mechanism of some TCMs has been studied through metabolomics (Liang et al., 2011). Metabolomics is also applied for the investigation on the toxicity of *Aconitum* alkaloids in animal models, and some carboxyl-containing metabolites (CCMs), such as amino acids (AAs) (Sun et al., 2009, 2014), tricarboxylic acid cycle intermediates (TCAs) (Sun et al., 2014), and fatty acid (FA) metabolism products (Cai et al., 2013) are found to be related to toxicity. It seems to indicate that CCMs might play an important role in the perturbation of metabolic profiles of *Aconitum* alkaloids poisoning. As usually known, there are many kinds of CCMs, e.g., AAs, TCAs, FAs, bile acids, and so on, which widely exist in human body and play vital physiological functions. For example, TCA cycle is the central energy metabolism pathway that connects carbohydrate, fat, and protein metabolism. Apart from the basic functions of AAs for the synthesis of protein, nucleotide and cell growth, and FAs for cell structural components, they are also contributed to the metabolite changes caused by *Aconitum* alkaloid poisoning (Dong et al., 2012; Cai et al., 2013; Jia et al., 2016). Therefore, it is urgent to comprehensively understand the CCMs profiling of poisoning caused by CW alkaloids. However, the detection of these components, especially FAs in serum, is obstructed by the low contents, high concentration variation, huge polarity difference, and matrix interference. Fortunately, 5-(diisopropylamino)amylamin (DIAAA)-derivatization coupled with ultrahigh performance liquid chromatography quadrupole-time-of-flight mass spectrometry (UHPLC-Q-TOF/MS) approach has been established for the detection of global CCMs in our laboratory (Bian et al., 2018). In this research, this approach was applied to illuminate the perturbed CCMs profiles after poisoning in the clinic for the first time. At the same time, the contents of diterpenoid alkaloids in the sera of CW poisoned patients were also quantified by dynamic multiple reaction monitoring (MRM) mode. It was the first time to systematically interpret the CW poisoning in humans. And also, the results from both quantification and metabolomics may provide some information for the clinicians to deal with CW poisoned patients.

## Materials and Methods

### Chemicals and Reagents

Carboxyl-containing metabolites standards, such as amino acid mixtures, TCA cycle intermediates, and short-chain fatty acids (SCFAs) were purchased from Sigma-Aldrich Laboratories, Inc., (St. Louis, MO, United States). Other fatty acid (FA) standards mixtures were purchased from Cayman Chemical (Ann Arbor, MI, United States). The detailed information on CCMs was shown in Supplementary Material.

DIAAA, O-(7-azabenzotriazol-1-yl)-N,N,N,N-tetramethyl-uronium hexafluorophosphate (HATU), 1-hydroxybenzotriazole hydrate (HOBt), triethylamine (TEA), and dimethyl sulfoxide (DMSO) (MS grade) were also bought from Sigma-Aldrich Laboratories, Inc., MS-grade acetonitrile and methanol were purchased from Anaqua Chemicals Supply (Houston, TX, United States) and MS-grade formic acid was provided by Sigma-Aldrich Laboratories, Inc., Deionized water was supplied by a Millipore water purification system (Millipore, United States).

### Patient Samples

The serum samples of nine healthy human and 11 CW poisoned patients obtained from First Affiliated Hospital of Kunming Medical University were recruited into the present study.

### UHPLC–QQQ/MS Quantitative Analysis of CW Alkaloids in Serum

The concentration of *Aconitum* alkaloids in the sera was determined using Agilent 1290 UHPLC coupled with 6490 triple quadrupole (QQQ) mass spectrometer (Santa Clara, CA, United States). The detailed sample preparation, LC condition, parameters of mass spectrometry (Supplementary Table S1), and the chemical structures (Supplementary Figure S1) of detected compounds were shown in Supplementary Material.

### Derivatization of Serum Samples

A total of 50 μL of serum was first mixed with four volumes of cold methanol to remove the proteins by centrifugation at 13000 rpm for 5 min at 4°C. The extraction was repeated three times and the combined supernatants were dried under a nitrogen stream. The residue was stored at -20°C prior to derivatization.

The derivatization was processed according to our previous method with little revision (Bian et al., 2018). First, HOBt and HATU were separately dissolved in DMSO at the concentration of 20 mM, while the DIAAA-TEA solution was prepared by dissolving 100 μmol of DIAAA and 200 μmol of TEA in 1 mL of DMSO. Thereafter, the dried residue of real samples or standards were sequentially mixed with 5 μL of HOBt, 5 μL of DIAAA-TEA solution, and 5 μL of HATU, followed by 1 min incubation at room temperature. Finally, 35 μL of acetonitrile was added to make up to the final volume of 50 μL, and 1 μL was directly injected into UHPLC-Q-TOF/MS.

### UHPLC-Q-TOF/MS Analysis of Endogenous CCMs

The separation for metabolic profiles of serum was performed on Agilent 1290 UHPLC system with Waters HSS T3 column (2.1 × 100 mm, 1.8 μm). The mass spectrometry was conducted on Agilent 6550 UHD accurate-mass Q-TOF/MS system with a dual Jet stream electrospray ion source (dual AJS ESI). The MS instrument was operated in positive (POS) ion mode. And the detailed information for LC condition (Supplementary Table S2), parameters of Q-TOF/MS was described in Supplementary Material.

### Data Processing and Analysis

The raw data were collected using Agilent MassHunter Workstation Software (Santa Clara, CA, United States). Molecular Feature Extractor was used to extract and detect molecular features with identical elution profiles, e.g., pseudomolecular ions, retention time. These data were saved as “.cef” files and then processed using Mass Profiler Professional software (Agilent Technologies) for compound alignment. The aligned data was imported into SIMCA-P software package (version 15.0, Umetrics, Umea, Sweden) for multivariate analysis in order to find the important metabolites, which can explain the differences of different sample groups. Then, the multivariate pattern recognition analysis, including unsupervised principal component analysis (PCA) and supervised orthogonal partial least squares-discriminant analysis (OPLS-DA), were constructed to assess the distributions of samples. S-plots and variable importance in projection (VIP) values were calculated to visualize the relationship between covariance and correlation within OPLS-DA result. Variables with significant contributions to discrimination were considered as potential biomarkers and subjected to further identification of the molecular formula. Metabolite peaks were assigned by MS/MS analysis or interpreted with available biochemical databases, such as HMDB^1^, Lipid Maps^2^, and METLIN^3^. Furthermore, the metabolites with statistical significance between groups were also measured by Student’s *t*-test using GraphPad Prism 5.0 (La Jolla, CA, United States) and receiver operating curves (ROC) using SPSS 17.0 (IBM, United States). To shed light on the metabolite networks and disturbed metabolic pathway of CW poisoning, the pathway analysis was applied by MetaboAnalyst 3.0^4^.

## Results

### Design of Experiments

**Figure 1:**
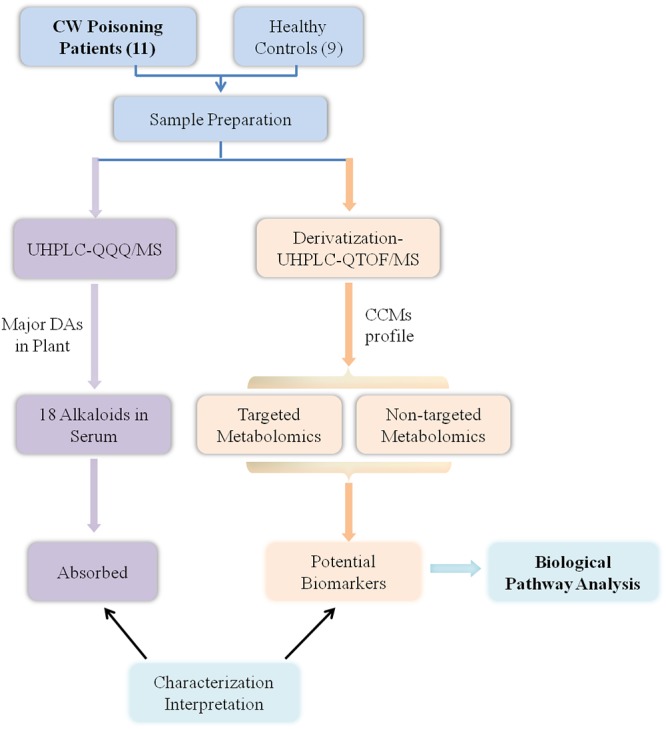
Workflow.

The workflow of this study was summarized in Figure 1. First, *Aconitum* alkaloids in the sera were detected and quantified using UHPLC-QQQ/MS. At the same time, the endogenous metabolites in the sera were also analyzed by UHPLC-Q-TOF/MS after DIAAA-derivatization. Then, CCMs were identified by comparison with the corresponding standards and the significance between healthy human and poisoning patients was pointed out by Student’s *t*-test. Moreover, to obtain more information on the changes of endogenous components after poisoning, the non-targeted metabolomics analysis coupled with pattern recognition was also conducted. Finally, potential biomarkers with significant difference from both targeted and non-targeted methods were used for metabolic pathway analysis.

### Clinical Symptoms of CW Poisoning Patients

Eleven patients were recruited into this study. The clinical symptoms of patients mainly include nervous system symptoms, cardiovascular system symptoms and digestive system symptoms. Symptoms of the nervous system include perioral numbness, dizziness, severe pain, parasympathetic excitation, and muscle weakness. Clinical diagnostic criteria include consumption of aconite, clinical symptoms and laboratory examinations.

### *Aconitum* Alkaloids Analysis in CW Poisoning Patients

**Figure 2:**
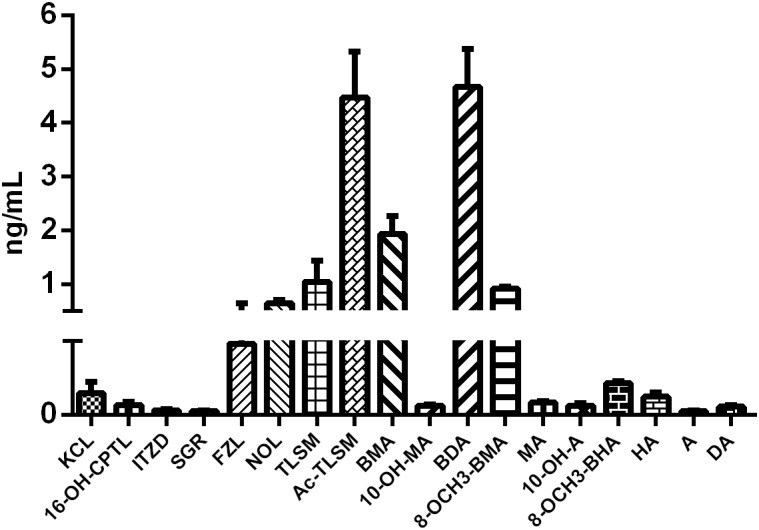
Serum concentration of *Aconitum* alkaloids in CW poisoning patients.

Eighteen *Aconitum* alkaloids, including 6 diester-diterpenoid alkaloids (DDAs), 4 monoester-diterpenoid alkaloids (MDAs), and 8 amine-diterpenoid alkaloids, were determined and quantified in CW poisoning patients by comparison with corresponding standards (Figure 2), while they were not detected in healthy human sera. Although DDAs, especially A, MA, and HA, are reported to be the main components account for the toxicity of *Aconitum* species (Liu et al., 2010), their contents in the sera of CW poisoning patients were relatively low at 0.086, 0.120, 0.031 ng/mL for MA, HA, and A, respectively. Another three DDAs, deoxyaconitine (DA), 10-OH-aconitine (10-OH-A), and 10-OH-mesaconitine (10-OH-MA), were also observed and the contents in the poisoning sera were 0.055, 0.060, and 0.062 ng/mL. Four MDAs, BMA, BDA, benzoyl-8-OCH_3_-mesaconine (8-OCH_3_-BMA), and benzoyl-8-OCH_3_-hypaconine (8-OCH_3_-BHA), had relatively high concentrations at 1.932, 4.665, 0.916, and 0.215 ng/mL, respectively. Amine-diterpenoid alkaloids, e.g., fuziline (FZL), neoline (NOL), talatizamine (TLSM), monoacetyltalatizamine (Ac-TLSM), were also detected with high contents in patients’ sera.

### Targeted Metabolomics Analysis

In order to sensitively determine the variations of potential biomarkers in poisoning patients, 69 CCMs standards belonging to five different types were firstly applied for the optimization of UHPLC-Q-TOF/MS approach. They were 35 polyunsaturated fatty acids (PUFAs), 20 AAs, 5 SCFAs, 8 TCAs and 1 pyruvate metabolites (Supplementary Table S3). As shown in Supplementary Figure S2, the endogenous metabolites in poisoning patients and healthy human could be separated well and annotated by comparison with corresponding standards. Then, the peak area ratios of targeted compounds to the internal standards were calculated for the *t*-test analysis. Two PUFAs, arachidonic acid and 12(S)-HETE, were detected in the sera and significantly increased after poisoning (*p* < 0.05). Sixteen AAs were observed in the sera (Supplementary Table S4), and 10 AAs significantly changed in the CW poisoning patients (Table 1). Alanine (Ala), threonine (Thr), proline (Pro), glutamic acid (Glu), glutamine (Gln), isoleucine (Ile), leucine (Leu), and phenylalanine (Phe) increased in the sera of CW poisoning patients, while valine (Val) and lysine (Lys) were decreased. Although five SCFAs were all found, only valeric acid was decreased with significance in patients. Three TCAs, including malic acid (Mal), succinic acid (Suc), and fumaric acid (Fum), and one pyruvate metabolite, lactic acid (Lac), were observed, and among them, Mal and Lac evidently increased after poisoning. The detailed information on these changed CCMs after poisoning was shown in Table 1.

**Table 1:** Identified differential metabolites in the serum of healthy controls and CW poisoning patients with targeted standards analysis and non-targeted metabolomic profile analysis.

No.	Identification	Sig	Fold change	VIP value	AUC	Sensitivity (%)	Specificity (%)	RT (min)	Metabolic pathway
1	Alanine^a^	^∗∗^	1.78	–	0.85	100.0	66.7	1.22	Aminoacyl-tRNA biosynthesis; Ala, Asp, and Glu metabolism
2	Threonine^a^	^∗∗∗^	3.93	–	0.97	81.8	100	2.10	Aminoacyl-tRNA biosynthesis; Val, Leu, and Ile biosynthesis
3	Valine^a^	^∗∗^	-1.48	–	0.90	88.9	90.9	2.35	Aminoacyl-tRNA biosynthesis
4	Proline^a^	^∗∗^	3.17	–	0.98	90.9	100.0	2.43	Aminoacyl-tRNA biosynthesis; Arg and Pro metabolism
5	Glutamic acid^a^	^∗^	9.51	1.23	0.94	73.7	100.0	4.18	Aminoacyl-tRNA biosynthesis; Gln and Glu metabolism
6	Glutamine^a^	^∗^	1.71	–	0.86	90.9	77.8	5.60	Aminoacyl-tRNA biosynthesis; Gln and Glu metabolism
7	Isoleucine^a^	^∗∗∗^	2.18	–	0.90	72.7	100.0	5.90	Aminoacyl-tRNA biosynthesis
8	Lysine^a^	^∗∗∗^	-1.82	1.24	0.94	90.9	88.9	5.90	Aminoacyl-tRNA biosynthesis; Lysine metabolism
9	Leucine^a^	^∗∗∗^	3.73	1.42	1.00	100.0	100.0	6.30	Aminoacyl-tRNA biosynthesis
10	Phenylalanine^a^	^∗∗∗^	4.84	1.80	0.98	100.0	88.9	9.00	Aminoacyl-tRNA biosynthesis; Phenylalanine metabolism
11	Malic acid^a^	^∗∗∗^	5.10	–	0.97	90.9	100.0	4.20	TCA cycle; Pyruvate metabolism
12	Lactic acid^a^	^∗^	1.51	4.70	0.77	90.9	55.6	4.70	Propanoate metabolism; Pyruvate metabolism
13	Hydroxybutyric acid isomer	^∗^	1.94	1.61	0.81	90.7	66.7	7.50	Propanoate metabolism; Butanoate metabolism
14	Homovanillic acid isomer	^∗^	2.00	–	0.84	72.70	88.9	13.20	Tyrosine metabolism
15	Homovanillic acid^a^	^∗^	90.93	1.81	0.96	81.8	100.0	14.10	Tyrosine metabolism
16	Valeric acid^a^	^∗^	-1.32	–	0.82	100.0	63.6	15.80	Fatty acid metabolism
17	Hydroxysebacic acid	^∗∗^	-30.12	1.76	0.83	66.7	100.0	17.30	Fatty acid metabolism
18	Hydroxyundecanedioic acid	^∗∗^	-35.33	1.97	0.83	66.7	100.0	18.40	Fatty acid metabolism
19	Hydroxydodecanedioic acid	^∗∗^	-25.12	3.47	0.82	77.8	100.0	19.80	Fatty acid metabolism
20	Hydroxydodecanedioic acid isomer	^∗∗^	-28.37	2.68	0.84	77.8	100.0	20.10	Fatty acid metabolism
21	Hydroxytetradecanedioic acid	^∗∗^	-58.43	3.89	0.99	90.9	100.0	23.60	Fatty acid metabolism
22	Lauric acid	^∗^	-1.47	2.64	0.80	88.9	72.7	35.55	Fatty acid biosynthesis
23	Hexadecasphinganine	^∗∗∗^	5.78	3.56	1.00	100.0	100.0	31.60	Sphingolipid metabolism
24	Sphinganine	^∗∗∗^	9.01	2.32	1.00	100.0	100.0	36.20	Sphingolipid metabolism
25	Eicosasphinganine	^∗∗∗^	10.12	1.93	1.00	100.0	100.0	38.90	Sphingolipid metabolism
26	12(S)-HETE^a^	^∗^	16.48	–	0.86	100.0	77.8	36.60	Arachidonic acid metabolism
27	Arachidonic acid^a^	^∗∗^	1.64	3.07	0.83	100.0	66.7	39.70	Arachidonic acid metabolism
28	Linolenic acid	^∗^	1.64	2.37	0.75	63.6	88.9	38.90	Alpha-linolenic acid metabolism
29	LysoPC(16:0)	^∗^	-1.22	1.10	0.70	45.5	88.9	39.90	Glycerophospholipid metabolism
30	Unidentified-1	^∗∗∗^	12.87	1.60	1.00	100.0	100.0	22.60	–
31	Unidentified-2	^∗∗∗^	36.98	3.49	1.00	100.0	100.0	33.00	–
32	Unidentified-3	^∗∗^	-40.25	1.48	0.83	66.7	100.0	36.80	–

*Fold change was the ratio of mean relative amount between the poisoning patients and controls; ^*a*^means metabolites that were identified with the standard by comparing with the exact mass data, MS fragmentation, and retention time; Sig was defined as ^∗∗∗^*p* < 0.001; ^∗∗^0.001 < *p* < 0.01; ^∗^0.01 < *p* < 0.05*.

### Non-targeted Metabolomic Profile Analysis

**Figure 3:**
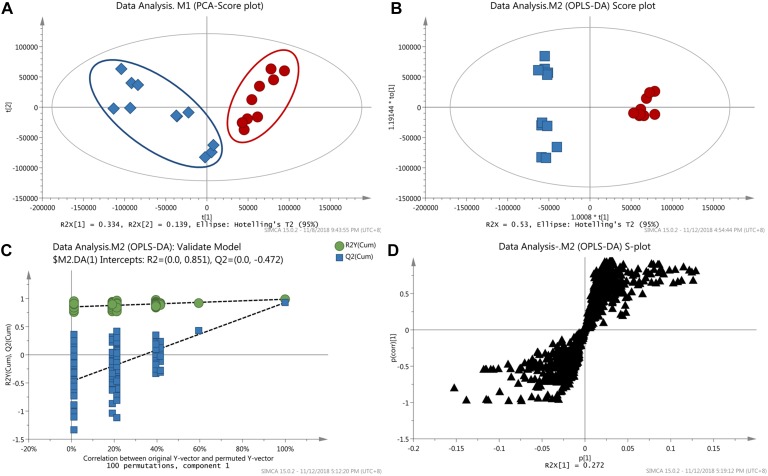
Results of metabolomics analysis. **(A)** PCA score plot discriminating the sera of CW poisoning patients from healthy controls (Red cycle: healthy control, Blue square: CW poisoning patients); **(B)** OPLS-DA score plot separating poisoning patients from healthy controls; **(C)** Result of permutation test with 100 times; **(D)** Loading S-plot of CW poisoning patients vs. healthy controls (Each triangle in the S-plot represents an ion).

Although above-mentioned targeted metabolomics analysis made the detection of objective metabolites more easy and sensitive, the important variables might be missed when they were not targets. Thus, the non-targeted metabolomic analysis was further conducted in this study. Prior to multivariate statistical analysis, the missing values in data matrix were processed according to the 80% rule (Smilde et al., 2005), and then the data sets were normalized using Pareto scaling by SIMCA-P. To visualize the subtle similarities and differences between the complex data sets, unsupervised PCA was first used to assess the metabolic phenotypes. According to the PCA model (*R*^2^X = 0.65, *Q*^2^X = 0.39), CW poisoning patients could separate well with the healthy controls (Figure 3A), suggesting that the metabolic profiles were different.

Next, supervised OPLS-DA was used to discriminate between poisoned patients and healthy controls, visualize the relationship between covariance and correlation, and further obtain variables that had significant contributions to discrimination. In this study, OPLS-DA model (1 + 3 + 0, *R*^2^X = 0.53, *R*^2^Y = 1, *Q*^2^ = 0.93, Figure 3B) further confirmed the differentiation of PCA results. With seven-fold cross-validation and 100 random permutation tests (Figure 3C), a valid model with good sensitivity and specificity was well established. In the corresponding S-plot (Figure 3D), each triangle represented a possible metabolite, and those far away from the origin with high magnitude and reliability were considered as potential biomarkers which had the greater contribution to differentiation. VIP value could also be used for separation, and here a threshold of 1.0 was set for identifying metabolites. Combining the results of S- and VIP-plots from OPLS-DA, potential variables responsible for this differentiation were characterized. And the changed metabolites were further confirmed by removing the adduct ions, [2M+H]^+^ ion, side-products formed by derivatization. Major discriminatory metabolites were shown in Table 1 and the identification of these metabolites was described in the following section.

### Biomarker Characterization andElucidation

Because each statistical analysis provided slightly different insight into high-dimensional data, student’s *t*-test and ROC were also performed to eliminate false discovery rates. ROC analysis was widely considered as the most objective and statistically valid method for biomarker performance evaluation. The area under the ROC (AUC) was used to evaluate classification performance. Fold change values of metabolites between two groups were also calculated by the ratios of integral non-overlapping areas of each metabolite. Metabolites with *p*-value <0.05, AUC > 0.7, and fold change >1.5 or <-1.5 were accepted as significance. Finally, 32 metabolites were considered responsible for CW poisoning (Table 1).

**Figure 4:**
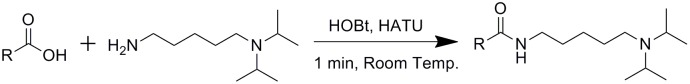
Derivatization of carboxyl-containing metabolites with DIAAA.

Next, the metabolites with significant differences were elucidated by the characteristic MS/MS fragmentation patterns and comparison of MS with that in the database, including Lipid Maps and METLIN as follows. Within a reasonable degree of measurement error (<5 ppm), the potential element composition (formula) was first obtained by the pseudomolecular ion, isotope ions, and adducts ions. As shown in Figure 4, DIAAA derivatization for carboxylic acids forms an amide. Thus, the molecular formula of corresponding carboxylic acid could be calculated by subtracting the formula of DIAAA (C_11_H_26_N_2_) and adding H_2_O. Furthermore, DIAAA derivatives were found to have characteristic MS/MS fragmentation ions using the standards, such as [M+H-42]^+^, [M+H-84]^+^, and/or [M+H-101]^+^, which were derived from the neutral loss of one or two propene and/or diisopropylamine. And, the ions at *m/z* 128 and 86 assigned to N-isopropyl-N-vinylpropan-2-aminium and N-vinylpropan-2-aminium were also related to the derivatization reagent DIAAA. As a result, the metabolite with these special fragmentation ions might contain the carboxyl group before DIAAA-derivatization. For example, metabolite 27 (Table 1, RT = 39.7 min) displayed the quasi-molecular ion at *m/z* 473.4469 in MS spectrum, indicating the formula of C_31_H_56_N_2_O with -1.04 ppm difference. The product ions at *m/z* 431.3994, 389.3535, 372.3256 were produced from the neutral loss of one or two propene and diisopropylamine (Supplementary Figure S3A). And distinctive MS/MS ions (*m/z* 128 and 86) were also found, which further confirmed it was a DIAAA derivative. By subtracting the formula of DIAAA (C_11_H_26_N_2_) and adding H_2_O, the formula of this compound before derivatization was determined to be C_20_H_32_O_2_, i.e., arachidonic acid by comparison with the corresponding standard. The fragmentation ions of compound 44 (Supplementary Table S4) at *m/z* 231.2075, 189.1603, 172.1339, 128.1441, and 86.0968 indicated it was DIAAA derivatized carboxylic acid. The pair of diagnostic ions at *m/z* 172.1339/154.1232 exhibited the presence of hydroxyl group, and the MS and the fragmentation ions at *m/z* 112.0760 and 69.0707 were also consistent with that of 3-hydroxybutyric acid derivative, which finally resulted in the structural identification (Supplementary Figure S3B). Similarly, compound 15 (Table 1) showed the [M+H]^+^ ion at *m/z* 351.2653 in MS spectrum, and the fragmentation ions at *m/z* 309.2173, 269.1709, 250.1441, 128.1439, and 86.0972 suggested that it was DIAAA-derivative. In addition, from the fragmentation ions at *m/z* 194.0830, 137.0606 and by comparison with the standard, it was identified as homovanillic acid (Supplementary Figure S3C). Based on the retention times, MS, and MS/MS spectra, a total of 53 CCMs (Supplementary Table S4) were identified and 27 of them had significant difference between healthy and poisoning patients.

At the same time, some non-derivatized metabolites were also found from the S- and VIP-plots. Compound 29 (Table 1) at RT 39.9 min exhibited an accurate [M+H]^+^ ion at *m/z* 496.3395, corresponding to the molecular formula C_24_H_50_NO_7_P. No DIAAA related fragmentation ions were observed. The major MS/MS fragmentation ions at *m/z* 184.0738 and 104.1076 were assigned to the fragmentations of [H_2_O_3_POCH_2_CH_2_N(CH_3_)_3_]^+^ and [HOCH_2_CH_2_N(CH_3_)_3_]^+^, respectively, which were clearly related to the hydrophilic head of the phosphatidylcholine (PC) class. Other fragmentation ions could also be well interpreted, like [M+H-H_2_O]^+^ at *m/z* 478.3299, [M+H-N(CH_3_)_3_]^+^ at *m/z* 419.2565, [M+H-C_5_H_13_NO_4_P]^+^ at *m/z* 313.2746 (Supplementary Figure S4). Comparing with HMDB and previous reports (Zhang et al., 2012; Liu et al., 2013), it was identified as LysoPC(16:0). Non-derivatized metabolites 23, 24 and 25 (Table 1) should be analogs from their similar MS/MS fragmentation pattern, like the fragmentation ions at *m/z* 106.1, 88.1, or [M+H-H_2_O]^+^. Finally, they were determined to be C_16_ sphinganine (hexadecasphinganine), sphinganine, and C_20_ sphinganine, (eicosasphinganine), respectively (Dong et al., 2015; Lee et al., 2017; Sun et al., 2017).

### Biological Pathway Analysis With MetaboAnalyst

The identified differential metabolites between healthy controls and CW poisoning patients in Table 1 were combined for pathway analysis using MetaboAnalyst 3.0. Based on existing database, MetaboAnalyst could give a visualization system with the most relevant pathways (Supplementary Figure S5). The result revealed that CW primarily disturbed aminoacyl-tRNA biosynthesis, Ala, aspartic acid (Asp) and Glu metabolism, Gln and Glu metabolism, arginine (Arg) and Pro metabolism, and pyruvate metabolism in human (impact value 0.1, significance 0.05), as shown in rats. Although the perturbed metabolism, like sphingolipid metabolism, alpha-linolenic acid metabolism, Phe metabolism, Lys degradation, and arachidonic acid metabolism were not significant after poisoning, they all had a high impact value on x axis, which meant that the changed metabolites in these pathways may also played key role in the poisoning.

## Discussion

As mentioned in the introduction, CW was widely used as a Chinese medicine or a tonic in China due to its pharmacological activities (Chan, 2014). However, because of the improper processing or overdose, at least several CW poisoning cases have been reported every year. Until now, no clinical index or biomarker could be applied for the diagnosis of *Aconitum* alkaloids poisoning, while some studies have been conducted in animal models. In this research, 11 poisoning patients, who consumed the soups made by CW and meats, were recruited into study. Both of *Aconitum* alkaloids and endogenous metabolites in patients and healthy humans were determined and compared to reveal the potential toxicity mechanism and find out the possible biomarkers for CW poisoning. To the best of our knowledge, this is the first report on the toxicological mechanisms for CW poisoning in clinic.

Firstly, the alkaloids in the sera of patients and healthy humans were determined by UHPLC-QQQ/MS. DA, 10-OH-MA and 10-OH-A, belonging to DDAs, were also detected in the sera of patients, except for three common toxic DDAs, MA, HA, and A. The LD_50_s for DA, 10-OH-A and 10-OH-MA were 1.90, 0.22, and 0.42 mg/kg in mouse intravenously, respectively, which were close or slightly higher than that of MA, HA, and A (0.10, 0.47, and 0.13 mg/kg) (ChemIDplus database). It indicated that these toxic DDAs in CW should be responsible for the intoxication cases. Although the toxicities of MDAs were weaker than that of DDAs, the contents of some MDAs in patients were relatively high, such as BMA, BDA, and 8-OCH_3_-BMA (Figure 2), which suggested that these MDAs might also contribute to the intoxication. In the newest edition of Chinese Pharmacopeia (CP) 2015, only three DDAs (MA, HA, and A) and three MDAs (BMA, BHA, and BA) were quantified for quality control of processed CW. Apparently, from our results and other previous clinical intoxication cases (Niitsu et al., 2013; Chan, 2015), the quantification of only these six compounds was still not enough for safety usage of *Aconitum* species herbs.

On the other hand, the contents of toxic DDAs in CW patients (0.086, 0.125, 0.031, 0.055, 0.062, and 0.060 ng/mL for MA, HA, A, DA, 10-OH-MA, and 10-OH-A) were found to be relatively lower than that of reported aconite poisoning cases, which varied from 0.1 to 259.5 ng/mL (Niitsu et al., 2013). This might be one of reasons that all patients in our study survived after CW poisoning. Of course, the low concentration might also be caused by many other factors, for example, the sampling times, low contents of toxic alkaloids in CW, metabolism and excretion in human body (Zhang et al., 2016).

To overcome the poor specificity of traditional metabolomic, which may result in the overlook of metabolites with low contents, DIAAA derivatization-UHPLC-Q-TOF/MS approach was applied in this research. Previous researches in animal models revealed that CCMs might be related to the toxicity of *Aconitum* alkaloids. Moreover, the sensitivities of CCMs in LC-MS analysis could be dramatically increased up to 2000 times after DIAAA derivatization (Bian et al., 2018). Therefore, the method specific to CCMs profiling was introduced to the elucidation of toxicological mechanisms of CW with a novel point of view.

The metabolomics can reflect the metabolites changes of organisms from terminal symptoms, thus has brought enormous opportunities for improving detection of toxicity and biomarker discovery. In our study, only 69 CCM standards for targeted metabolomics are not quite enough, thus non-targeted metabolomics was also applied to hunt for much more metabolites, which were related to the toxicity of *Aconitum* alkaloids. Finally, a total of 58 metabolites (Supplementary Table S4), including 28 targeted CCMs and 30 non-targeted metabolites were detected. Among them, 32 metabolites, containing 10 AAs, 8 organic acids, 5 FAs, 3 sphingolipids, 1 TCA cycle intermediate, 1 pyruvate metabolite, 1 PC, and 3 unidentified metabolites (Table 1), were found to have the significant difference after CW poisoning.

**Figure 5:**
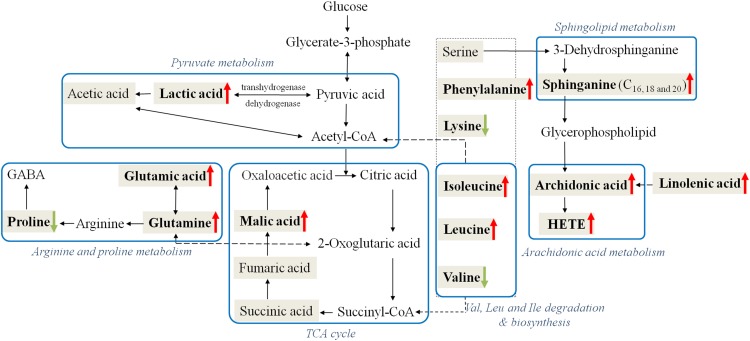
Perturbed metabolic regulatory network in response to CW poisoning. Metabolites in bold are denoted as with significant variation after CW poisoning (Red arrow symbols were up-regulated, green were down-regulated) and metabolites in the shadowed cell are also detected in our research while with no significant change. Note that the blue rectangular strips represent the related metabolic pathways.

The perturbed metabolites were related to aminoacyl-tRNA biosynthesis, Glu and Gln metabolism, pyruvate metabolism and several correlate pathways (Supplementary Figure S5). Moreover, these metabolic pathways were correlated with each other, and a metabolic network of affected pathways was thus formed in body. Considering potential linkages, the correlation networks of the potential biomarkers in response to CW poisoning were described in Figure 5.

**Figure 6:**
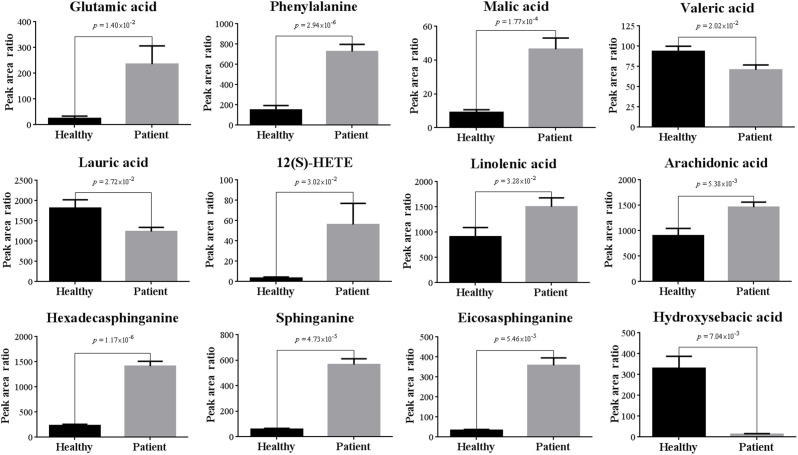
Comparisons of representative metabolites in serum of CW poisoning patients.

Ten out of 16 detected AAs were significantly changed after poisoning. Most of them were up-regulated, and the increased level of Glu and Phe were up to 9.5 and 4.8 times (Table 1 and Figure 6). Some up-regulated AAs can also serve as energy storage unit (such as Pro, Ile, and Leu) (Zuo et al., 2018). Thus, the increased levels of these AAs in serum suggested that CW poisoning can modulate energy metabolism in the human body. The increased energy requirement after poisoning might be the main reason for the up-regulation of most AAs in serum. In addition, Mal was also increased in our study (Figure 6). It is well known that TCA cycle is one of the most important energy metabolism in the human body, where it is a major source of adenosine triphosphate (ATPs) production. The increased levels of Mal in the poisoning patients further confirmed the imbalanced energy metabolism.

Five FAs [valeric acid, lauric acid, 12(S)-HETE, linolenic acid, and arachidonic acid] were significantly changed after CW intoxication, and they primarily involved in arachidonic acid metabolism, alpha-linolenic acid metabolism, and FA metabolism, etc., (Supplementary Figure S5) Among them, the levels of three PUFAs were up-regulated after poisoning, particularly, the content of 12(S)-HETE was increased even by 16 times (Table 1 and Figure 6). However, another two FAs, valeric acid and lauric acid (Table 1 and Figure 6), displayed declining tendencies, suggesting the abnormal FA metabolism was caused by poisoning. According to the previous study (Cai et al., 2013), the abnormal peroxidation of PUFAs could damage the myocardial mitochondria, thus leading to heart arrhythmia. Although the mechanism is still unknown, this might be another reason for its poisoning. Additionally, FAs are known to represent the major source of energy production (by β-oxidation) and energy storage in humans. Our results also indicated the existence of energy metabolism disturbance after poisoning. Three markedly elevated sphingolipids suggested that sphingolipid metabolism was disturbed (Table 1). Sphingomyelinases can hydrolyze sphingomyelin to create a train of bioactive lipids such as sphingosine (Cai et al., 2013). The up-regulation of sphingomyelinases might be responsible for the accumulation of sphingosine.

Some dicarboxylic acids were found to tremendously decrease after CW poisoning, for example, hydroxysebacic acid could be barely found in the sera of poisoned patients (Figure 6). These dicarboxylic acids were possibly associated with the endoplasmic reticulum in liver or kidney cells, and the increasing rates of β-oxidation may induce the reduction of dicarboxylic acids in the sera of CW poisoning patients (Mortensen and Gregersen, 1981; Draye et al., 1988). This indicated that FAs oxidation might also be disturbed by CW poisoning. This is the first report on the decline of dicarboxylic acid metabolites in *Aconitum* alkaloids intoxication cases.

## Conclusion

In summary, clinical CW intoxication was first systematically investigated from both of alkaloids quantification and endogenous metabolites variations. Except for the common toxic diterpenoid alkaloids, DA, 10-OH-A, 10-OH-MA, BDA, and 8-OCH_3_-BMA with high toxicities were also detected in the sera of poisoning patients. Considering the wide use of the products of *Aconitum* species, like Fuzi, Heishunpian, Baifupian, etc., it is urgent to establish a more suitable quality control method for the safe use. At the same time, the DIAAA derivatization-UHPLC-Q-TOF/MS approach was applied in targeted and non-targeted metabolomics analysis, which provided higher sensitivity for CCMs. As a result, 32 altered metabolites after poisoning were identified. Among them, HETEs, and some dicarboxylic acids were first found to be related to *Aconitum* alkaloids toxicity. Finally, the biological pathway analysis disclosed that the metabolic pathways of amino acid metabolism, TCA cycle, pyruvate metabolism, FA metabolism, sphingolipid metabolism, and arachidonic acid metabolism were associated with CW poisoning. These results not only can provide more information on the mechanism of CW intoxication but also help the clinical diagnosis of CW poisoning.

## Data Availability

All datasets generated for this study are included in the manuscript and/or the Supplementary Files.

## Ethics Statement

The study was carried out in accordance with the principles of the Declaration of Helsinki and First Affiliated Hospital of Kunming Medical University guidelines and approved by the Ethics Committee of First Affiliated Hospital of Kunming Medical University. Prior to any study procedure, all human participants gave written informed consent.

## Author Contributions

NL and J-LW designed the experiments and revised the manuscript. HW and JY collected the serum samples and made the clinical examination of patients. YZ performed the main experiments and drafted the manuscript. XB developed the DIAAA derivatization-UHPLC-Q-TOF/MS approach and assisted the identification of metabolites. All authors approved the final version to be published.

## Conflict of Interest Statement

The authors declare that the research was conducted in the absence of any commercial or financial relationships that could be construed as a potential conflict of interest.

## Footnotes

1 http://www.hmdb.ca/

2 http://www.lipidmaps.org/

3 https://metlin.scripps.edu/index.php

4 http://www.metaboanalyst.ca/MetaboAnalyst/

## Abbreviations

A: aconitine
AA: amino acid
BA: benzoylaconitine
BDA: benzoyldeoxyaconine
BHA: benzoylhypaconitine
BMA: benzoylmesaconine
CCM: carboxyl-containing metabolite
CW: the root of *Aconitum kusnezoffii*, Caowu in Chinese
DA: deoxyaconine
DDA: diester diterpenoid alkaloid
DIAAA: 5-(diisopropylamino)amylamine
FA: fatty acid
HA: hypaconitine
HATU: *O*-(7-azabenzotriazol-1-yl)-*N*,*N*,*N′*,*N′*-tetramethyluronium hexafluorophosphate
HETE: hydroxyeicosatetraenoic acid
HOBt: 1-hydroxybenzotriazole hydrate
LCFA: long-chain fatty acid
MA: mesaconitine
MDA: monoester diterpenoid alkaloid
OPLS-DA: orthogonal projections to latent structures discriminant analysis
PCA: principal component analysis
ROC: receiver operating curve
SCFA: short-chain fatty acid
TCM: traditional Chinese medicine
TEA: triethylamine
UHPLC–QQQ: ultrahigh performance liquid chromatography-triple quadrupole
UHPLC-Q-TOF/MS: ultrahigh performance liquid chromatography-quadrupole-time of flight mass spectrometry
VIP: variable importance in projection.
