# Supplementary material for: Metabolomics of Clinical Poisoning by Aconitum Alkaloids Using Derivatization LC-MS
Source: Front Pharmacol. 2019 Mar 22;10:275. doi: 10.3389/fphar.2019.00275 (PMC6439482; doi:10.3389/fphar.2019.00275)
Supplement: Supplementary file 1 [file Table_1.docx]

**Supplementary Materials for**

**Metabolomics of Clinical Poisoning by *Aconitum* Alkaloids using derivatization LC-MS**

Yida Zhang^a^, Xiqing Bian^a^, Jing Yang^b^, Haiying Wu^b,^*, Jian-Lin Wu^a ,^*, and Na Li^a,^*

^a^ State Key Laboratory of Quality Research in Chinese Medicine, Macau Institute for Applied Research in Medicine and Health, Macau University of Science and Technology, Macao, China

^b^ Department of Emergency, The First Affiliated Hospital of Kunming Medical University, Kunming, Yunnan 650000, P.R. China.

*Correspondence:

Haiying Wu 874705340@qq.com

Jian-Lin Wu jlwu@must.edu.mo

Na Li nli@must.edu.mo

**Supplementary Materials includes:**

**Chemicals**

**Methods**

**Figure S1** Chemical structures of 18 *Aconitum* alkaloids determined in CW poisoning patients

**Figure S2** Total compound chromatogram (TCC) of CW poisoning patient **(A)**, healthy control **(B)** and standards **(C)**

**Figure S3** MS/MS spectra of arachidonic acid **(A)**, 3-hydroxybutyric acid **(B)**, and homovanillic acid **(C)** derivatized with DIAAA

**Figure S4** MS/MS spectrum of non-derivatized metabolite, LysoPC(16:0)

**Figure S5** Perturbed metabolic pathways after CW poisoning. **1**. Aminoacyl-tRNA biosynthesis; **2**. Glutamine and glutamate metabolism; **3**. Alanine, aspartate and glutamate metabolism; **4**. Pyruvate metabolism; **5**. Arginine and proline metabolism; **6**. Arachidonic acid metabolism; **7**. alpha-Linolenic acid metabolism; **8**. Sphingolipid metabolism (pathways with high pathway impact and/or high p-value)

**Table S1** Optimized dynamic MRM parameters for each alkaloid in UHPLC–QQQ/MS quantitative analysis

**Table S2** LC gradient elution for UHPLC-Q-TOF/MS analysis of serum sample after derivatization

**Table S3** Derivatized standards used in targeted standards analysis

**Table S4** Identified metabolites in serum using UHPLC-Q-TOF/MS analysis

**Chemicals**

Some carboxyl-containing metabolite standards were purchased from Cayman Chemical (Ann Arbor, MI). They are (S)-hydroxyeicosatetraenoic acid (HETE) HPLC mixture containing 5(S)-, 8(S)-, 11(S)-, 12(S)-, and 15(S)-HETE; hydroperoxy HPLC mixture containing 5(S)-, 12(S)-, and 15(S)-hydroperoxyeicosatetraenoic acid (HPETE), 9(S)- and 13(S)-hydroperoxyoctadecadienoic acid (HPODE); ω-3 hydroxy acid HPLC mixture containing 5(S)-, 12(S)-, and 15(S)-hydroxyeicosapentaenoic acid (HEPE), 13(S)-hydroxyoctadecatrienoic acid (HOTrE), and 15(S)-Hydroxyeicosatrienoic acid (HETrE); cyclopentenone prostaglandin (PG) HPLC mixture containing PGA2, PGB2, PGD2, PGE2 and PGJ2; prostaglandin HPLC mixture containing PGE1, PGE2, PGF1α, 6-keto PGF1α and PGF2α; vasoactive eicosanoid HPLC mixture containing thromboxane B2 (TXB2), 11-dehydro TXB2, 6-keto PGF1α, 2,3-dinor-6-keto PGF1α and 12(S)-hydroxyheptadecatrienoic acid (HHTrE); prostaglandin metabolite HPLC mixture containing 13,14-dihydro-15-keto PGD2 (DK-PGD2), 13,14-dihydro-15-keto PGE2 (DK-PGE2), 11β-PGF2α, 13,14-dihydro-15-keto PGF2α (DK-PGF2α) and PGF2α; Leukotriene B4 (LTB4), arachidonic acid; amino acids mixtures containing L-alanine (Ala), arginine (Arg), aspartic acid (Asp), glutamic acid (Glu), glycine (Gly), histidine (His), isoleucine (Ile), leucine (Leu), lysine (Lys), methionine (Met), phenylalanine (Phe), proline (Pro), serine (Ser), threonine (Thr), tyrosine (Tyr), tryptophan (Trp), glutamine (Gln) and valine (Val); TCA cycle intermediates containing aconitic acid, malic acid (Mal), fumaric acid (Fum), α-ketoglutaric acid (α-KG), lactic acid (Lac), succinic acid (Suc), oxaloacetic acid (OAA), isocitric acid (Isocit), citric acid (Cit); short-chain fatty acids containing butyric acid, isobutyric acid, propionic acid, valeric acid and isovaleric acid were purchased from Sigma-Aldrich Laboratories, Inc. (St. Louis, MO). The internal standard 12(S)-HETE-*d*_8_ was provided by Cayman Chemical (Ann Arbor, MI), 4-Cl-phenylalanine was obtained from Sigma-Aldrich Laboratories, Inc. (St. Louis, MO).

**Methods**

**1. UHPLC–QQQ Quantitative Analysis of CW Alkaloids in Serum**

**1.1 Sample Preparation**

Serum samples (100 μL) were thawed and spiked with 10 μL internal standard solution (Tetrandrine, 200 ng/mL). The mixture was deproteinized and extracted with 500 μL acetonitrile and vortexed for 5 min. After centrifuged at 14,000 rpm for 10 min at 4°C, the supernatant was transferred to another tube and evaporated to dryness under a stream of nitrogen. Afterwards, the residue was redissolved in 100 μL 50% acetonitrile, vortexed for 2 min followed by centrifugation at 14,000 rpm at 4 °C for 10 min. An aliquot of 2 μL supernatant was injected into the UHPLC-QQQ/MS for analysis.

**1.2 UHPLC-QQQ/MS Analysis**

Quantitative analysis was conducted on Agilent 1290 UHPLC system (Agilent Technologies, Santa Clara, CA, USA). Chromatographic separation was performed on a Waters ACQUITY UPLC® BEH C18 column (2.1×100 mm, 1.8 μm). Gradient elution was achieved using the mobile phase consisting of water (A) and acetonitrile (B) with 0.1% formic acid in each with the following schedules: 8% of B at 0–0.5 min, 8–20% of B at 0.5–5 min, 20–30% of B at 5–5.5 min, 30–35% of B at 5.5–12 min, 35–95% B at 12–13 min, 95% B at 13–16 min and then equilibrated with 8% B for 4 min. The column temperature was set at 30 °C, and the flow rate was 0.3 mL/min.

Quantitative analysis was carried out using an Agilent 6490 QQQ mass spectrometer (Santa Clara, CA, USA). After optimization, the Jet Stream parameters were set at the temperature of 300 °C and flow rate of 10 L/min for sheath gas and ESI was run at positive ion mode with capillary voltage of 4500 V, nozzle voltage of 300 V, nebulizer pressure of 23 psi, and 13 L/min and 250 °C for drying gas. Two segments scan and dynamic multiple reactions monitoring (MRM) mode were adopted to monitor the transitions of analytes separately for maximal sensitivity. The first segment (0-0.5 min) directly flowed into waste to get rid of influence on MS. The optimized parameters such as characteristic transitions (precursor ion → product ion), fragmentors, retention times and collision energy values selected for the target analytes and IS were shown in **Table S1**.

**2. UHPLC-Q-TOF/MS Analysis of Endogenous CCMs**

The separation for metabolic profiles of serum was performed on Agilent 1290 UHPLC system with Waters HSS T3 column (2.1 × 100 mm, 1.8 μm). Chromatographic separation was performed using the mobile phase consisting of water (A) and acetonitrile (B) with 0.1% formic acid in each. And the LC gradient was showed in **Table S2**. The column temperature was maintained at 40 °C and the autosampler was set at 4 °C. And the flow rate was set at 0.3 mL/min.

The mass spectrometry was conducted on an Agilent 6550 UHD accurate-mass Q-TOF/MS system with a dual Jet stream electrospray ion source (dual AJS ESI). The instrument was operated in positive full scan mode, and mass spectrum was recorded across the range *m/z* 100−1500 with accurate mass measurement of all peaks. Parameters for the Jet Stream technology included a superheated nitrogen sheath gas temperature of 300 °C and a flow rate of 11 L/min. The MS parameters were set as: drying gas flow 15 L/min, gas temperature 250 °C, nebulizer pressure 20 psi, capillary voltage 5000 V, nozzle voltage 500 V, and fragmentor voltage 380 V. A low flow of TOF reference mixture was nebulized for continuous calibration in positive ion mode: *m/z* 121.0509 (C_5_H_4_N_4_) and 922.0098 (C_18_H_18_F_24_N_3_O_6_P_3_). Automated MS/MS and targeted MS/MS were applied for MS/MS acquisition with collision cell energy 30 eV.

**FIGURE S1**︱Chemical structures of 18 *Aconitum* alkaloids determined in CW poisoning patients


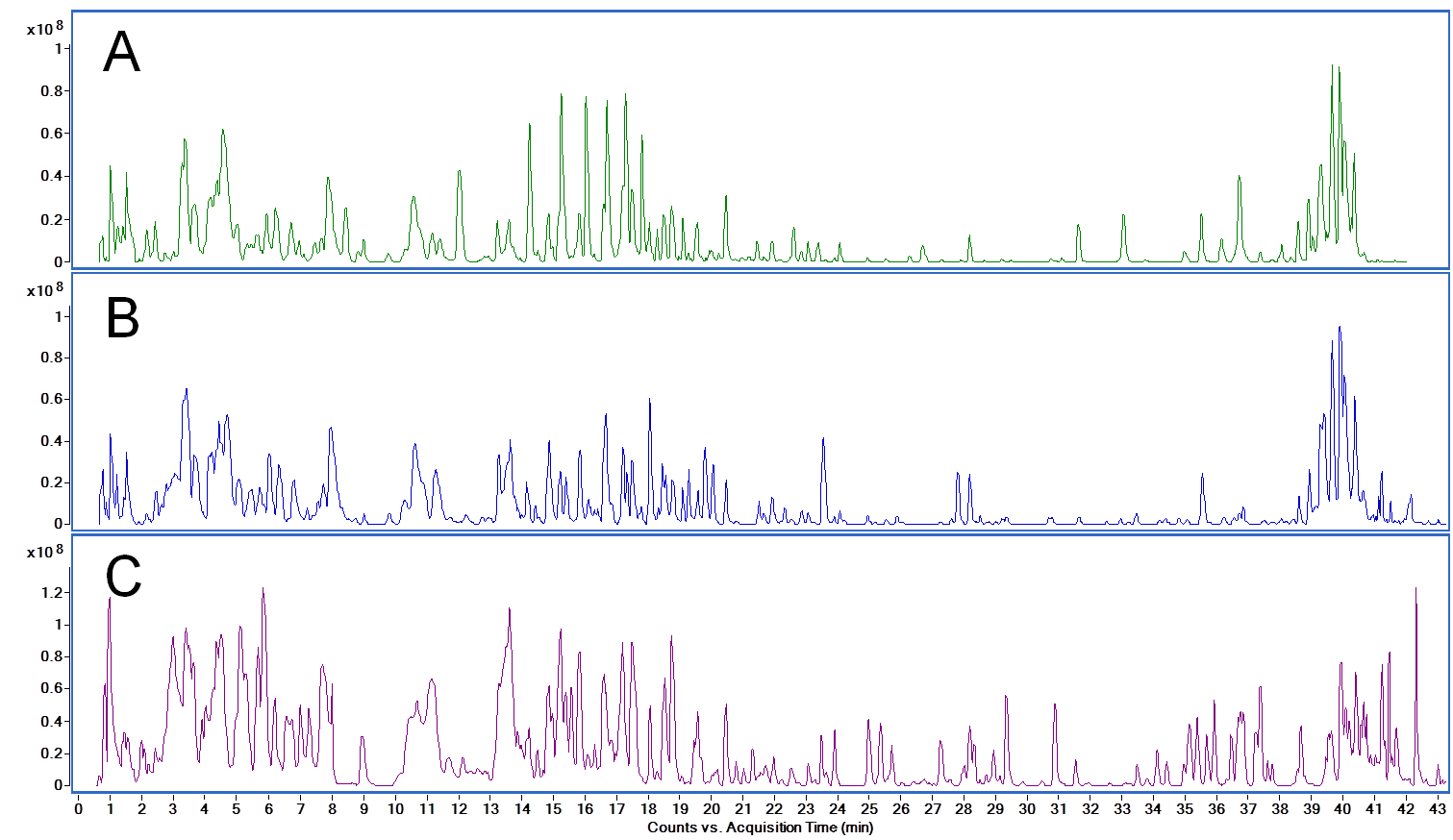


**FIGURE S2**︱Total compound chromatogram (TCC) of CW poisoning patient **(A)**, healthy control **(B)** and standards **(C)**

**
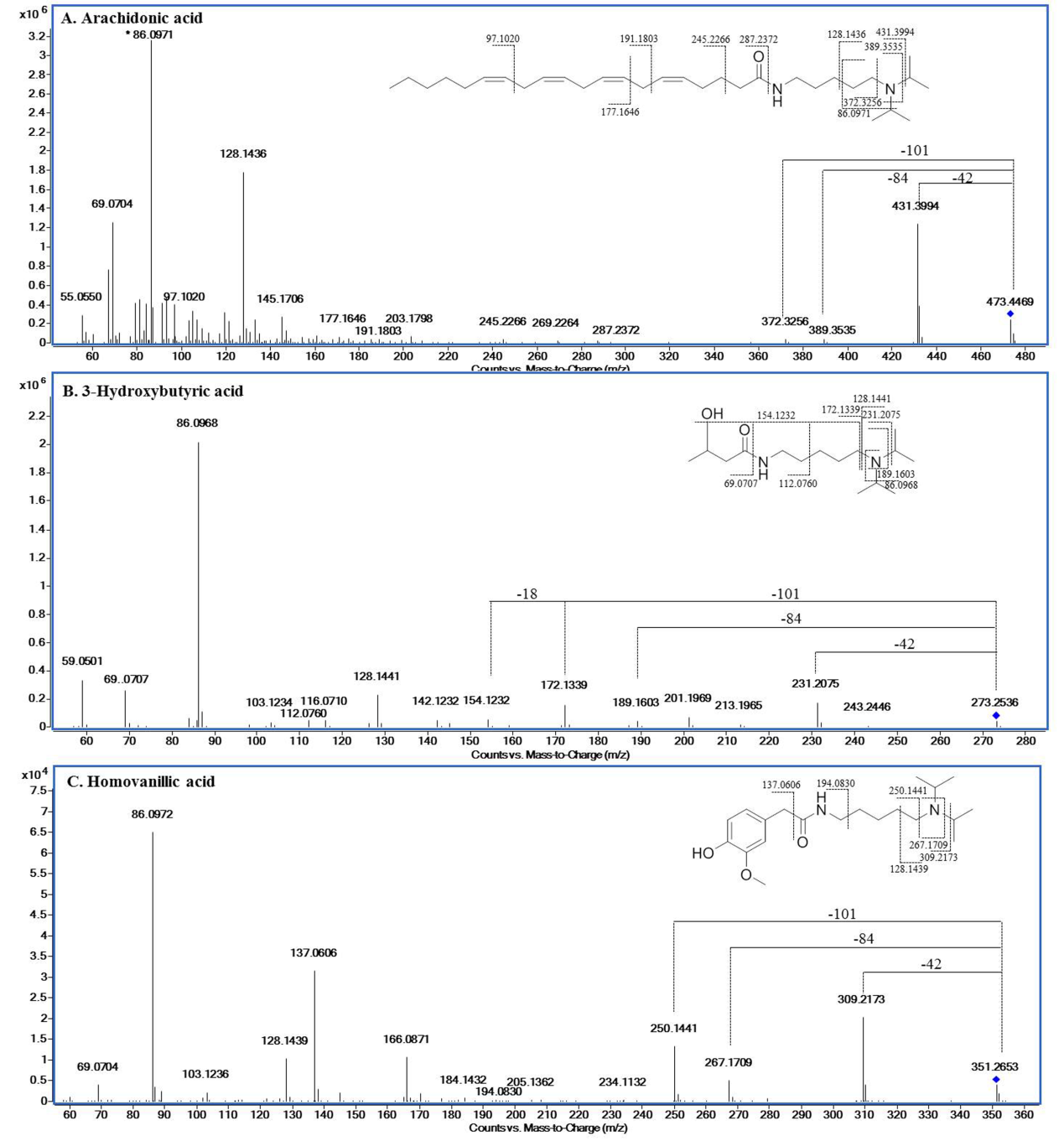
**

**FIGURE S3**︱MS/MS spectra of arachidonic acid **(A)**, 3-hydroxybutyric acid **(B)**, and homovanillic acid **(C)** derivatized with DIAAA

**
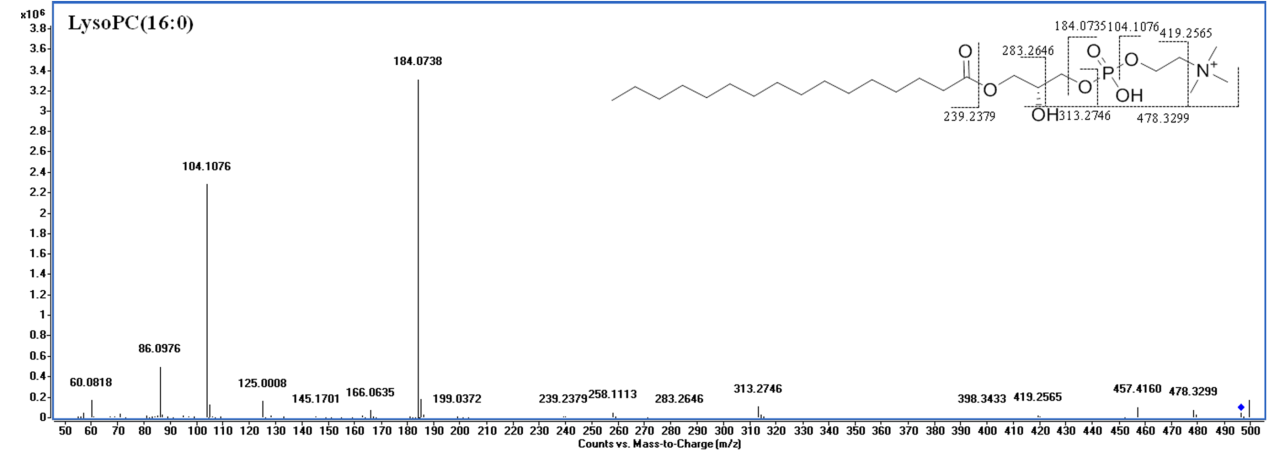
**

**FIGURE S4**︱MS/MS spectrum of non-derivatized metabolite, LysoPC(16:0)

**
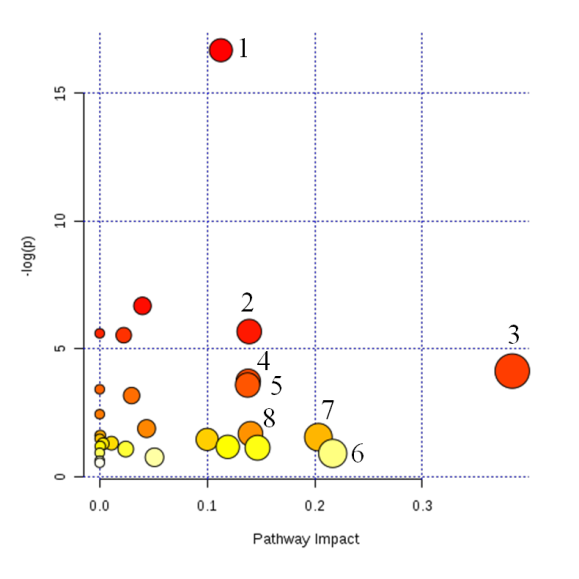
**

**FIGURE S5**︱Perturbed metabolic pathways after CW poisoning. **1**. Aminoacyl-tRNA biosynthesis; **2**. Glutamine and glutamate metabolism; **3**. Alanine, aspartate and glutamate metabolism; **4**. Pyruvate metabolism; **5**. Arginine and proline metabolism; **6**. Arachidonic acid metabolism; **7**. alpha-Linolenic acid metabolism; **8**. Sphingolipid metabolism (pathways with high pathway impact and/or high p-value)

**TABLE S1**︱Optimized dynamic MRM parameters for each alkaloid in UHPLC–QQQ/MS quantitative analysis

| No. | Analytes | Abbreviation | Formula | Transitions | Precursor | Product | Fragmentor | Collision | RT |
| --- | --- | --- | --- | --- | --- | --- | --- | --- | --- |
|  |  |  |  |  | ion (*m/z*) | ion (*m/z*) | (V) | energy (eV) | (min) |
| 1 | Karakoline | KCL | C_22_H_35_NO_4_ | Target | 378.3 | 360.3 | 207 | 32 | 2.5 |
|  |  |  |  | Qualifier | 378.3 | 58.2 | 207 | 48 |  |
| 2 | 16β-Hydroxycardiopetaline | 16-OH-CPTL | C_21_H_33_NO_4_ | Target | 364.2 | 346.3 | 192 | 32 | 2.8 |
|  |  |  |  | Qualifier | 364.2 | 58.3 | 192 | 48 |  |
| 3 | Isotalatizidine | ITZD | C_23_H_37_NO_5_ | Target | 408.3 | 390.3 | 178 | 28 | 3.2 |
|  |  |  |  | Qualifier | 408.3 | 58.2 | 178 | 48 |  |
| 4 | Songorine | SGR | C_22_H_31_NO_3_ | Target | 358.2 | 340.3 | 162 | 24 | 3.6 |
|  |  |  |  | Qualifier | 358.2 | 91.1 | 162 | 72 |  |
| 5 | Fuziline | FZL | C_24_H_39_NO_7_ | Target | 454.3 | 436.3 | 198 | 32 | 4.3 |
|  |  |  |  | Qualifier | 454.3 | 58.2 | 198 | 52 |  |
| 6 | Neoline | NOL | C_24_H_39_NO_6_ | Target | 438.3 | 420.3 | 188 | 28 | 4.6 |
|  |  |  |  | Qualifier | 438.3 | 58.2 | 188 | 44 |  |
| 7 | Talatizamine | TLSM | C_24_H_39_NO_5_ | Target | 422.3 | 390.3 | 178 | 28 | 5.4 |
|  |  |  |  | Qualifier | 422.3 | 58.2 | 178 | 44 |  |
| 8 | Monoacetyltalatizamine | Ac-TLSM | C_26_H_41_NO_6_ | Target | 464.3 | 432.3 | 183 | 28 | 6.8 |
|  |  |  |  | Qualifier | 464.3 | 58.2 | 183 | 44 |  |
| 9 | Benzoyl-8-OH-mesaconine | BMA | C_31_H_43_NO_10_ | Target | 590.3 | 105.1 | 217 | 60 | 7.4 |
|  |  |  |  | Qualifier | 590.3 | 77.2 | 217 | 100 |  |
| 10 | 10-OH-mesaconitine | 10-OH-MA | C_33_H_45_NO_12_ | Target | 648.3 | 105.1 | 222 | 68 | 8.7 |
|  |  |  |  | Qualifier | 648.3 | 77.2 | 222 | 100 |  |
| 11 | Benzoyldeoxyaconine | BDA | C_32_H_45_NO_9_ | Target | 588.3 | 556.3 | 224 | 32 | 8.8 |
|  |  |  |  | Qualifier | 588.3 | 105.1 | 224 | 60 |  |
| 12 | Benzoyl-8-OCH3-mesaconine | 8-MeO-BMA | C_32_H_45_NO_10_ | Target | 604.3 | 105.1 | 227 | 56 | 8.9 |
|  |  |  |  | Qualifier | 604.3 | 77.2 | 227 | 100 |  |
| 13 | Mesaconitine | MA | C_33_H_45_NO_11_ | Target | 632.3 | 572.3 | 234 | 32 | 9.8 |
|  |  |  |  | Qualifier | 632.3 | 105.1 | 234 | 68 |  |
| 14 | 10-OH-aconitine | 10-OH-A | C_34_H_47_NO_12_ | Target | 662.3 | 105.1 | 234 | 68 | 10 |
|  |  |  |  | Qualifier | 662.3 | 602.3 | 234 | 32 |  |
| 15 | 8-O-methyl-benzoylhypaconine | 8-MeO-BHA | C_32_H_45_NO_9_ | Target | 588.3 | 105.1 | 230 | 52 | 10.4 |
|  |  |  |  | Qualifier | 588.3 | 77.2 | 230 | 100 |  |
| 16 | Hypoaconitine | HA | C_33_H_45_NO_10_ | Target | 616.3 | 556.3 | 208 | 32 | 11.3 |
|  |  |  |  | Qualifier | 616.3 | 105.1 | 208 | 68 |  |
| 17 | Aconitine | A | C_34_H_47_NO_11_ | Target | 646.3 | 586.3 | 200 | 32 | 11.4 |
|  |  |  |  | Qualifier | 646.3 | 105.1 | 200 | 68 |  |
| 18 | Deoxyaconitine | DA | C_34_H_47_NO_10_ | Target | 630.3 | 570.3 | 229 | 32 | 13.2 |
|  |  |  |  | Qualifier | 630.3 | 105.1 | 229 | 68 |  |

**TABLE S2**︱ LC gradient elution for UHPLC-Q-TOF/MS analysis of serum sample after derivatization

| **Total time(min)** | **A(%)** | **B(%)** | **Flow rate (mL/min)** |
| --- | --- | --- | --- |
| 0 | 95 | 5 | 0.3 |
| 0.5 | 95 | 5 | 0.3 |
| 2.5 | 94 | 6 | 0.3 |
| 3.5 | 93 | 7 | 0.3 |
| 4.3 | 92.7 | 7.3 | 0.3 |
| 7.3 | 92.2 | 7.8 | 0.3 |
| 10 | 91 | 9 | 0.3 |
| 12 | 86 | 14 | 0.3 |
| 17 | 77 | 23 | 0.3 |
| 18 | 75 | 25 | 0.3 |
| 25 | 67 | 33 | 0.3 |
| 25.5 | 65 | 35 | 0.3 |
| 33.5 | 53 | 47 | 0.3 |
| 37 | 40 | 60 | 0.3 |
| 39 | 5 | 95 | 0.3 |
| 42.9 | 5 | 95 | 0.3 |
| 43 | 98 | 5 | 0.3 |

**TABLE S3**︱Derivatized standards used in targeted standards analysis

| **Classification** | **Name** | **Formula** | **Derivatization formula** | [M+H]^+^ |
| --- | --- | --- | --- | --- |
| **PUFAs** | Arachidonoic acid | C_20_H_32_O_2_ | C_31_H_56_N_2_O | 473.4465 |
|  | LTB4 | C_20_H_32_O_4_ | C_31_H_56_N_2_O_3_ | 505.4364 |
|  | 12(S)-HPETE | C_20_H_32_O_4_ | C_31_H_56_N_2_O_3_ | 505.4364 |
|  | 15(S)-HPETE | C_20_H_32_O_4_ | C_31_H_56_N_2_O_3_ | 505.4364 |
|  | 5(S)-HPETE | C_20_H_32_O_4_ | C_31_H_56_N_2_O_3_ | 505.4364 |
|  | 13(S)-HPODE | C_18_H_32_O_4_ | C_29_H_56_N_2_O_3_ | 481.4364 |
|  | 9(S)-HPODE | C_18_H_32_O_4_ | C_29_H_56_N_2_O_3_ | 481.4364 |
|  | 15(S)-HETE | C_20_H_32_O_3_ | C_31_H_56_N_2_O_2_ | 489.4415 |
|  | 11(S)-HETE | C_20_H_32_O_3_ | C_31_H_56_N_2_O_2_ | 489.4415 |
|  | 12(S)-HETE | C_20_H_32_O_3_ | C_31_H_56_N_2_O_2_ | 489.4415 |
|  | 8(S)-HETE | C_20_H_32_O_3_ | C_31_H_56_N_2_O_2_ | 489.4415 |
|  | 5(S)-HETE | C_20_H_32_O_3_ | C_31_H_56_N_2_O_2_ | 489.4415 |
|  | 15(S)-HEPE | C_20_H_30_O_3_ | C_31_H_54_N_2_O_2_ | 487.4258 |
|  | 12(S)-HEPE | C_20_H_30_O_3_ | C_31_H_54_N_2_O_2_ | 487.4258 |
|  | 5(S)-HEPE | C_20_H_30_O_3_ | C_31_H_54_N_2_O_2_ | 487.4258 |
|  | 13(S)-HOTrE | C_18_H_30_O_3_ | C_29_H_54_N_2_O_2_ | 463.4258 |
|  | 15(S)-HETrE | C_20_H_34_O_3_ | C_31_H_58_N_2_O_2_ | 491.4571 |
|  | PGA2 | C_20_H_30_O_4_ | C_31_H_54_N_2_O_3_ | 503.4207 |
|  | PGB2 | C_20_H_30_O_4_ | C_31_H_54_N_2_O_3_ | 503.4207 |
|  | PGJ2 | C_20_H_30_O_4_ | C_31_H_54_N_2_O_3_ | 503.4207 |
|  | PGE2 | C_20_H_32_O_5_ | C_31_H_56_N_2_O_4_ | 521.4313 |
|  | PGD2 | C_20_H_32_O_5_ | C_31_H_56_N_2_O_4_ | 521.4313 |
|  | 13, 14-dehydro-15-keto PGD2 | C_20_H_32_O_5_ | C_31_H_56_N_2_O_4_ | 521.4313 |
|  | 13, 14-dehydro-15-keto PGE2 | C_20_H_32_O_5_ | C_31_H_56_N_2_O_4_ | 521.4313 |
|  | 13, 14-dehydro-15-keto PGF2α | C_20_H_34_O_5_ | C_31_H_58_N_2_O_4_ | 523.4469 |
|  | 11β-PGF2α | C_20_H_34_O_5_ | C_31_H_58_N_2_O_4_ | 523.4469 |
|  | PGF2β | C_20_H_34_O_5_ | C_31_H_58_N_2_O_4_ | 523.4469 |
|  | PGF2α | C_20_H_34_O_5_ | C_31_H_58_N_2_O_4_ | 523.4469 |
|  | PGE1 | C_20_H_34_O_5_ | C_31_H_58_N_2_O_4_ | 523.4469 |
|  | PGF1α | C_20_H_36_O_5_ | C_31_H_60_N_2_O_4_ | 525.4626 |
|  | TXB2 | C_20_H_34_O_6_ | C_31_H_58_N_2_O_5_ | 539.4418 |
|  | 6-keto-PGF1α | C_20_H_34_O_6_ | C_31_H_58_N_2_O_5_ | 539.4418 |
|  | 2,3-dinor-6-keto-PGF1α | C_18_H_30_O_6_ | C_29_H_54_N_2_O_5_ | 511.4105 |
|  | 11-dehydro-TXB2 | C_20_H_32_O_6_ | C_31_H_56_N_2_O_5_ | 537.4262 |
|  | 12(S)-HHTrE | C_17_H_28_O_3_ | C_28_H_52_N_2_O_2_ | 449.4102 |
| **Amino acids** | Alanine (Ala) | C_3_H_7_NO_2_ | C_14_H_31_N_3_O | 258.2540 |
|  | Arginine (Arg) | C_6_H_14_N_4_O_2_ | C_17_H_38_N_6_O | 343.3180 |
|  | Aspartic acid (Asp) | C_4_H_7_NO_4_ | C_15_H_31_N_3_O_3_ | 302.2438 |
|  | Cysteine (Cys) | C_3_H_7_NO_2_S | C_14_H_31_N_3_OS | 290.2261 |
|  | Glutamic acid (Glu) | C_5_H_9_NO_4_ | C_16_H_33_N_3_O_3_ | 316.2595 |
|  | Glycine (Gly) | C_2_H_5_NO_2_ | C_13_H_29_N_3_O | 244.2383 |
|  | Histidine (His) | C_6_H_9_N_3_O_2_ | C_17_H_33_N_5_O | 324.2758 |
|  | Isoleucine (Ile) | C_6_H_13_NO_2_ | C_17_H_37_N_3_O | 300.3009 |
|  | Leucine (Leu) | C_6_H_13_NO_2_ | C_17_H_37_N_3_O | 300.3009 |
|  | Lysine (Lys) | C_6_H_14_N_2_O_2_ | C_17_H_38_N_4_O | 315.3129 |
|  | Methionine (Met) | C_5_H_11_NO_2_S | C_16_H_35_N_3_OS | 318.2574 |
|  | Phenylalanine (Phe) | C_9_H_11_NO_2_ | C_20_H_35_N_3_O | 334.2853 |
|  | Proline (Pro) | C_5_H_9_NO_2_ | C_16_H_33_N_3_O | 284.2696 |
|  | Serine (Ser) | C_3_H_7_NO_3_ | C_14_H_31_N_3_O_2_ | 274.2489 |
|  | Threonine (Thr) | C_4_H_9_NO_3_ | C_15_H_33_N_3_O_2_ | 288.2646 |
|  | Tryptophan (Trp) | C_11_H_12_N_2_O_2_ | C_22_H_36_N_4_O | 373.2962 |
|  | Tyrosine (Tyr) | C_9_H_11_NO_3_ | C_20_H_35_N_3_O_2_ | 350.2802 |
|  | Valine (Val) | C_5_H_11_NO_2_ | C_16_H_35_N_3_O | 286.2853 |
|  | Asparagine (Asn) | C_4_H_8_N_2_O_3_ | C_15_H_32_N_4_O_2_ | 301.2598 |
|  | Glutamine (Gln) | C_5_H_10_N_2_O_3_ | C_16_H_34_N_4_O_2_ | 315.2755 |
| **TCA intermediates** | Aconitic acid | C_6_H_6_O_6_ | C_17_H_30_N_2_O_5_ | 343.2227 |
|  | Oxaloacetic acid (OAA) | C_4_H_4_O_5_ | C_15_H_28_N_2_O_4_ | 301.2122 |
|  | Isocitric acid (Isocit) | C_6_H_8_O_7_ | C_17_H_32_N_2_O_6_ | 361.2333 |
|  | Citric acid (Cit) | C_6_H_8_O_7_ | C_17_H_32_N_2_O_6_ | 361.2333 |
|  | Malic acid (Mal) | C_4_H_6_O_5_ | C_15_H_30_N_2_O_4_ | 303.2278 |
|  | Fumaric acid (Fum) | C_4_H_4_O_4_ | C_15_H_28_N_2_O_3_ | 285.2173 |
|  | alpha-Ketoglutaric acid (α-KG) | C_5_H_6_O_5_ | C_16_H_30_N_2_O_4_ | 315.2278 |
|  | Succinic acid (Suc) | C_4_H_6_O_4_ | C_15_H_30_N_2_O_3_ | 287.2329 |
| **Pyruvate metabolites** | Lactic acid (Lac) | C_3_H_6_O_3_ | C_14_H_30_N_2_O_2_ | 259.2380 |
| **SCFAs** | Propionic acid | C_3_H_6_O_2_ | C_14_H_30_N_2_O | 243.2431 |
|  | Isobutyric acid | C_4_H_8_O_2_ | C_15_H_32_N_2_O | 257.2587 |
|  | Butyric acid | C_4_H_8_O_2_ | C_15_H_32_N_2_O | 257.2587 |
|  | Isovaleric acid | C_5_H_10_O_2_ | C_16_H_34_N_2_O | 271.2744 |
|  | Valeric acid | C_5_H_10_O_2_ | C_16_H_34_N_2_O | 271.2744 |

**TABLE S4**︱Identified metabolites in serum using UHPLC-Q-TOF/MS analysis

| **No.** | **Identification** | **RT (min)** | **Derivatization formula** | **[M+H]^+^** | **Formula** | **MS/MS** |
| --- | --- | --- | --- | --- | --- | --- |
|  |  |  |  |  |  |  |
| 1 | Alanine | 1.22 | C_14_H_32_N_3_O_2_ | 258.254 | C_3_H_7_NO_2_ | 216.2079, 174.1610, 157.1342, 128.1438, 86.0972 |
| 2 | Threonine^a^ | 2.10 | C_15_H_33_N_3_O_2_ | 288.2646 | C_4_H_9_NO_3_ | 246.2184, 228.2084, 169.1352, 152.1067, 128.1444, 86.0971 |
| 3 | Valine^a^ | 2.35 | C_16_H_35_N_3_O | 286.2864 | C_5_H_11_NO_2_ | 244.2384, 202.1845, 185.1652, 168.1643, 128.1438, 86.0971 |
| 4 | Proline^a^ | 2.43 | C_16_H_33_N_3_O | 284.2694 | C_5_H_9_NO_2_ | 242.2231, 200.1755, 183.1496, 168.1386, 128.1441, 86.0971 |
| 5 | Glutamic acid^a^ | 4.18 | C_16_H_33_N_3_O_3_ | 316.2595 | C_5_H_9_NO_4_ | 274.2127, 232.1655, 130.0501, 128.1443, 86.0971 |
| 6 | Glutamine^a^ | 5.60 | C_16_H_34_N_4_O_2_ | 315.2756 | C_5_H_10_N_2_O_3_ | 273.2285, 256.2044, 214.1558, 197.1301, 129.0680, 128.1443, 86.0971 |
| 7 | Isoleucine^a^ | 5.90 | C_17_H_37_N_3_O | 300.3011 | C_6_H_13_NO_2_ | 258.2542, 241.1941, 216.2073, 199.1808, 145.1702, 128.1436, 86.0971 |
| 8 | Lysine^a^ | 5.90 | C_17_H_38_N_4_O | 315.3120 | C_6_H_14_N_2_O_2_ | 273.266, 231.2167, 214.1971, 171.1502, 129.1028, 128.1441, 112.0762, 86.0971 |
| 9 | Leucine^a^ | 6.30 | C_17_H_37_N_3_O | 300.3011 | C_6_H_13_NO_2_ | 258.2547, 241.1943, 216.2102, 199.1812, 145.1705, 128.1438, 86.0973 |
| 10 | Phenylalanine^a^ | 9.00 | C_20_H_35_N_3_O | 334.2856 | C_9_H_11_NO_2_ | 292.2395, 250.1928, 233.1654, 128.1437, 86.0974 |
| 11 | Malic acid^a^ | 4.20 | C_15_H_30_N_2_O_4_ | 303.2282 | C_4_H_6_O_5_ | 261.1805, 219.1335, 201.1221, 184.0962, 166.0853, 145.1692, 128.1445, 86.0970 |
| 12 | Lactic acid^a^ | 4.70 | C_14_H_30_N_2_O_2_ | 259.2380 | C_3_H_6_O_3_ | 217.1918, 175.1441, 158.1178, 140.0998, 128.1439, 86.0971 |
| 13 | Hydroxybutyric acid isomer | 7.50 | C_15_H_32_N_2_O_2_ | 273.2550 | C_4_H_8_O_3_ | 231.2074, 189.1607, 172.1343, 128.1436, 86.0975 |
| 14 | Homovanillic acid isomer | 13.20 | C_20_H_34_N_2_O_3_ | 351.2641 | C_9_H_10_O_4_ | 309, 2174, 291.2044, 249.2038, 128.1439, 86.0971 |
| 15 | Homovanillic acid^a^ | 14.10 | C_20_H_34_N_2_O_3_ | 351.2641 | C_9_H_10_O_4_ | 309.2173, 267.1709, 250.1441, 194.0830, 137.0606, 128.1439, 86.0972 |
| 16 | Valeric acid^a^ | 15.80 | C_16_H_34_N_2_O | 271.2744 | C_5_H_10_O_2_ | 229.2284, 187.1811, 170.1547, 145.1706, 128.1441, 86.0973 |
| 17 | Hydroxysebacic acid | 17.30 | C_21_H_42_N_2_O_4_ | 387.3212 | C_10_H_18_O_5_ | 345.2752, 303.2270, 286.2022, 268.1913, 128.1440, 86.0970 |
| 18 | Hydroxyundecanedioic acid | 18.40 | C_22_H_44_N_2_O_4_ | 401.3375 | C_11_H_20_O_5_ | 359.2904, 317.2424, 300.2180, 282.2051, 214.1446, 128.1440, 86.0972 |
| 19 | Hydroxydodecanedioic acid | 19.80 | C_23_H_46_N_2_O_4_ | 415.3545 | C_12_H_22_O_5_ | 373.3057, 331.2586, 314.2338, 296.2218, 238.1807, 152.1074, 128.1440, 86.0972 |
| 20 | Hydroxydodecanedioic acid isomer | 20.10 | C_23_H_46_N_2_O_4_ | 415.3545 | C_12_H_22_O_5_ | 373.3069, 315.2641, 296.2225, 238.1810, 152.1076, 128.1442, 86.0973 |
| 21 | Hydroxytetradecanedioic acid | 23.60 | C_25_H_50_N_2_O_4_ | 443.3860 | C_14_H_26_O_5_ | 401.3369, 359.2902, 342.2646, 324.2531, 128.1441, 86.0973 |
| 22 | Lauric acid | 35.55 | C_23_H_48_N_2_O | 369.3839 | C_12_H_24_O_2_ | 327.3373, 285.0939, 268.2635, 183.1746, 128.1439, 86.0972 |
| 23 | Hexadecasphinganine | 31.60 | ­- | 274.2743 | C_16_H_35_NO_2_ | 256.264, 230.2484, 212.237, 172.8613, 106.0871, 88.0765, 70.066, 57.0706 |
| 24 | Sphinganine | 36.20 | ­- | 302.3058 | C_18_H_39_NO_2_ | 284.2955, 258.2794, 240.2680, 172.8644, 106.0870, 88.0763, 70.0661 |
| 25 | Eicosasphinganine | 38.90 | ­- | 330.3370 | C_20_H_43_NO_2_ | 312.3258, 286.3119, 268.3011, 184.0727, 106.0868, 88.0772 |
| 26 | 12(S)-HETE^a^ | 36.60 | C_31_H_56_N_2_O_2_ | 489.4415 | C_20_H_32_O_3_ | 471.4317, 429.3831, 287.3352, 370.3150, 285.2207, 133.0862, 128.1433, 89.0602 |
| 27 | Arachidonic acid^a^ | 39.70 | C_31_H_56_N_2_O | 473.4475 | C_20_H_32_O_2_ | 431.3994, 372.3256, 287.2372, 245.2266, 191.1803, 177.1646, 128.1436, 97.1020, 86.0971 |
| 28 | Linolenic acid | 38.90 | C_29_H_54_N_2_O | 447.4315 | C_18_H_30_O_2_ | 405.3839, 363.3364, 346.3102, 290.2475, 261.2234, 128.1449, 86.0967 |
| 29 | LysoPC(16:0) | 39.90 | ­- | 496.3435 | C_24_H_50_NO_7_P | 478.3299, 419.2565, 313.2746, 283.2646, 239.2379, 184.0735, 104.1076 |
| 30 | Unidentified-1 | 22.6 | C_26_H_48_N_2_O | 405.3837 | C_15_H_24_O_2_ | 363.7914, 304.2639, 219.1747, 187.2177, 161.1329, 128.1434, 86.0973 |
| 31 | Unidentified-2 | 33.00 | ­- | 387.1808 | C_22_H_26_O_6_ | 371.2269, 281.1023, 233.0818, 203.1073, 177.1161, 128.1439, 105.0703 |
| 32 | Unidentified-3 | 36.80 | C_39_H_74_N_2_O_8_ | 699.5518 | C_28_H_50_O_9_ | 657.5009, 615.1114, 238.1820, 184.0739, 128.1436, 86.0967 |
| 33 | Glycine^a^ | 1.10 | C_13_H_29_N_3_O | 244.2383 | C_2_H_5_NO_2_ | 202.1919, 160.1457, 143.1186, 128.1431, 86.0969 |
| 34 | Serine^a^ | 1.20 | C_14_H_31_N_3_O_2_ | 274.2489 | C_3_H_7_NO_3_ | 232.2087, 190.1531, 128.1428, 86.0969 |
| 35 | Methionine | 4.40 | C_16_H_35_N_3_OS | 318.2574 | C_5_H_11_NO_2_S | 276.2103, 234.1637, 217.1382, 128.1426, 86.0937 |
| 36 | Tyrosine^a^ | 4.50 | C_20_H_35_N_3_O_2_ | 350.2802 | C_9_H_11_NO_3_ | 308.2334, 291.1341, 249.1612, 204.1441, 128.1431, 86.0971 |
| 37 | Aspartic acid^a^ | 6.70 | C_15_H_31_N_3_O_3_ | 302.2446 | C_4_H_7_NO_4_ | 171.1493, 129.1021, 128.1439, 112.0763, 86.0963 |
| 38 | Tryptophan^a^ | 12.70 | C_22_H_36_N_4_O | 373.2962 | C_11_H_12_N_2_O_2_ | 356.2719, 331.2507, 314.2218, 272.1773, 255.1502, 227.1542, 170.0609, 128.1437, 86.0971 |
| 39 | Acetic acid | 4.50 | C_13_H_28_N_2_O | 229.23 | C_2_H_4_O_2_ | 187.1807, 145.1343, 128.1472, 86.0968 |
| 40 | Propionic acid | 6.80 | C_14_H_30_N_2_O | 243.2431 | C_3_H_6_O_2_ | 201.1990, 159.1507, 142.1233, 128.1438, 86.0969 |
| 41 | Isobutyric acid | 10.80 | C_15_H_32_N_2_O | 257.2587 | C_4_H_8_O_2_ | 215.2124, 171.1491, 156.1391, 128.1436, 86.0972 |
| 42 | Butyric acid | 11.40 | C_15_H_32_N_2_O | 257.2587 | C_4_H_8_O_2_ | 215.2129, 173.1274, 145.1731, 128.1436, 86.0972 |
| 43 | Isovaleric acid | 15.60 | C_15_H_32_N_2_O | 257.2587 | C_4_H_8_O_2_ | 229.2295, 187.1813, 170.1552, 145.1714, 128.1443, 86.0971 |
| 44 | 3-Hydroxybutyric acid^a^ | 5.40 | C_15_H_32_N_2_O_2_ | 273.2550 | C_4_H_8_O_3_ | 231.2075, 189.1603, 172.1339, 154.1232, 128.1441, 112.0709, 86.0973, 69.0702 |
| 45 | Aminomalonic acid | 4.30 | C_14_H_29_N_3_O_3_ | 288.2284 | C_3_H_5_NO_4_ | 213.1974, 171.1569, 129.1027, 128.1489, 86.0972 |
| 46 | Fumaric acid^a^ | 7.40 | C_15_H_28_N_2_O_3_ | 285.2173 | C_4_H_4_O_4_ | 243.1709, 201.1254, 184.0977, 170.1921, 128.1452, 86.0975 |
| 47 | Succinic acid | 5.30 | C_15_H_30_N_2_O_3_ | 287.2329 | C_4_H_6_O_4_ | 245.1886, 227.1753, 185.1308, 168.1033, 128.1441, 86.0963 |
| 48 | Azelaic acid or Nonate | 18.10 | C_20_H_40_N_2_O_3_ | 357.3112 | C_9_H_16_O_4_ | 357.3111, 315.2648, 297.2551, 256.1914, 238.1807, 213.1966, 171.1499, 152.1074, 128.1438, 86.0972 |
| 49 | Caproic acid | 18.90 | C_17_H_36_N_2_O | 285.29 | C_6_H_12_O_2_ | 243.2441, 201.1974, 184.1721, 170.1916, 128.1442, 86.0974 |
| 50 | Caprylic acid | 22.50 | C_19_H_40_N_2_O | 313.3214 | C_8_H_16_O_2_ | 271.2752, 229.2283, 212.2018, 128.1446, 86.0973 |
| 51 | Caprylic acid isomer | 25.03 | C_19_H_40_N_2_O | 313.3214 | C_8_H_16_O_2_ | 271.2747, 229.2275, 212.2014, 145.1705, 128.1439, 86.0971 |
| 52 | Capric acid | 30.80 | C_21_H_44_N_2_O | 341.3526 | C_10_H_20_O_2_ | 299.3061, 257.2616, 240.2315, 128.1437, 86.0963 |
| 53 | Myristic acid | 38.62 | C_25_H_52_N_2_O | 397.4152 | C_14_H_28_O_2_ | 355.3688, 313.3211, 296.2947, 211.2060, 128.1436, 86.0970 |
| 54 | Palmitic acid | 39.91 | C_27_H_56_N_2_O | 425.4465 | C_16_H_32_O_2_ | 383.4008, 341.3537, 324.3268, 128.1442, 86.0975 |
| 55 | Stearic acid | 40.38 | C_29_H_60_N_2_O | 453.4778 | C_18_H_36_O_2_ | 411.4318, 369.3851, 352.3585, 296.2991, 128.1444, 86.0975 |
| 56 | 15(S)-HETE | 35.76 | C_31_H_56_N_2_O_2_ | 489.4415 | C_20_H_32_O_3_ | 471.4305, 429.3833, 387.3377, 370.3090, 285.2232, 243.2097, 161.0993, 128.1435, 86.0936 |
| 57 | 11(S)-HETE | 36.33 | C_31_H_56_N_2_O_2_ | 489.4415 | C_20_H_32_O_3_ | 471.4311, 429.3837, 387.3347, 370.3099, 285.2255, 243.2138, 133.1011, 128.1434, 86.0969 |
| 58 | 5(S)-HETE | 37.30 | C_31_H_56_N_2_O_2_ | 489.4415 | C_20_H_32_O_3_ | 471.4303, 429.3835, 387.3310, 370.3095, 347.3061, 319.2779, 243.2118, 128.1441, 86.0971 |
